# Soleris^®^ Automated System for the Rapid Detection of *Burkholderia cepacia* Complex in Cosmetic Products

**DOI:** 10.1093/jaoacint/qsac109

**Published:** 2022-09-21

**Authors:** Lei Zhang, Jerry Tolan, Nicholas Lavigne, Carolyn Montei, Robert Donofrio, Preetha Biswas

**Affiliations:** Microbiology R&D, Neogen Corporation, 620 Lesher Place, Lansing, MI 48912, USA; Microbiology R&D, Neogen Corporation, 620 Lesher Place, Lansing, MI 48912, USA; Microbiology R&D, Neogen Corporation, 620 Lesher Place, Lansing, MI 48912, USA; Microbiology R&D, Neogen Corporation, 620 Lesher Place, Lansing, MI 48912, USA; Microbiology R&D, Neogen Corporation, 620 Lesher Place, Lansing, MI 48912, USA; Microbiology R&D, Neogen Corporation, 620 Lesher Place, Lansing, MI 48912, USA

## Abstract

**Background:**

*Burkholderia cepacia* complex (Bcc) has emerged as an important opportunistic pathogen with rising concern in pharmaceuticals and cosmetic products. The Bcc supplement (S2-BCC-S) was purposely developed and used with the *Pseudomonas* vial (PD-109) for the detection of Bcc through the Soleris^®^ Next Generation automated instrument system.

**Objective:**

This study aimed to evaluate the performance of the Soleris Bcc testing method for cosmetic products.

**Method:**

Inclusivity and exclusivity were assessed with the Soleris Bcc method and the United States Pharmacopeia (USP) method in three enrichment broths. Matrix testing was conducted using 28 cosmetic products to compare the equivalency of the Soleris Bcc method to that of the USP reference method. Repeatability of the Soleris Bcc assay, method robustness, product stability, and lot-to-lot consistency of the Soleris reagents were also assessed.

**Results:**

Both the Soleris Bcc and the USP methods supported the growth of all 26 inclusivity strains, except the USP method missed one inclusivity strain in one broth. For exclusivity, 0–6% was presumptive positive with the Soleris Bcc method, and 42–48% was presumptive positive with the reference method. Kappa index was 0.96 for the matrix testing, indicating a good agreement between the Soleris Bcc assay and the reference method for testing Bcc in cosmetics. Repeatability results showed the coefficient of variation was less than 4%. The robustness and ruggedness study yielded detection times within 1 h differences when small variations were introduced. The lot-to-lot study showed consistent results among four lots of the Bcc reagents.

**Conclusions:**

The automated Soleris method was successfully demonstrated to be robust, sensitive, and specific for Bcc detection in cosmetic products.

**Highlights:**

The Soleris Bcc method is user-friendly. It shows the results in real time and generates the report automatically. Implementation of this method for detection of Bcc in cosmetics would save significant time and resources.

*Burkholderia cepacia* is a Gram-negative, obligately aerobic, and rod-shaped bacterium, which was first isolated from rotting onions (1). Originally belonging to rRNA group II of the genus of *Pseudomonas*, DNA-DNA hybridization and 16S rRNA sequence alignment studies showed that they were sufficiently different from *Pseudomonas* species; thus *P. cepacia* and six other species were later transferred to the new genus *Burkholderia* in 1992 (2). *Burkholderia cepacia* was once thought to be a single species but has expanded to the *Burkholderia cepacia* complex (Bcc), comprising a group of closely related *Burkholderia* species that exhibit a high degree of 16S rRNA and *rec*A gene sequence similarity, and moderate levels of DNA–DNA hybridization (3, 4). Members of the Bcc are ubiquitous in nature and are widely found in soil, water, rhizosphere, and agricultural products (5). Remarkably, Bcc bacteria, such as *B. cepacia*, *B. multivorans*, and *B. cenocepacia*, can adapt to adverse conditions and remain viable under harsh conditions and can even use certain antimicrobials as carbon sources (5, 6). Studies have shown that Bcc strains remained viable in drinking water or in a saline solution with 0.05% benzalkonium chloride for a long time (7, 8). Bcc bacteria are resistant to many common antibiotics and are able to acquire resistance against many more antibiotics. Due to its ecological and metabolic versatility and resistance to a wide range of antibiotics and antiseptics, Bcc has emerged as an opportunistic pathogen of concern (9, 10). Bcc organisms cause serious infections in individuals with cystitis fibrosis and chronic granulomatous disease (11, 12). They also pose a high risk in mechanically ventilated patients, the immunosuppressed, infants, the elderly, and those with underlying disease conditions (9, 13).

In recent years, Bcc has been considered the most common microbial contaminant found in nonsterile pharmaceutical and personal care products. Multiple products have been recalled from the market due to contamination with this group of bacteria, such as disinfectant solutions, hospital soaps, nasal sprays, mouthwash, and anesthetics (5, 14–16), and numerous Bcc infections and outbreaks have been reported (17–23).

Due to its easy adaptation to adverse conditions, resistance to antimicrobial preservative systems, and risks to patients, the United States Pharmacopeia (USP) has created a new chapter USP <60> on December 1, 2019, to address the public health issues posed by Bcc organisms. USP <60>, titled “Microbiological Examination of Nonsterile Products—Tests for *Burkholderia cepacia* Complex,” contains test procedures and media formulations for the detection of Bcc organisms (24). The USP method for detection of Bcc requires 5–6 days for completion, including 48–72 h enrichment followed by 48–72 h incubation on selective agar plates.

The Soleris^®^ Next Generation automated system is a rapid microbial testing system designed to detect the target microorganisms in a variety of matrixes including foods, beverages, nutraceuticals, cosmetics, and toiletries. The system is based on real-time detection of color or fluorescence changes in growth media due to microbial metabolism. It includes a Soleris incubator instrument integrated with a secure software package and ready-to-use Soleris vial media and supplements. Soleris vials contain two zones, an incubation zone with selective broth for microbial growth and a separate reading window zone monitored spectrophotometrically by the instrument software.

The Soleris *Pseudomonas* vial (PD-109) supports the growth of *Pseudomonas* spp. The addition of the newly developed *B. cepacia* complex supplement (S2-BCC-S) can specifically detect Bcc organisms, while *Pseudomonas* and other closely related organisms, such as *Stenotrophomonas maltophilia*, are inhibited. The supplement is aqueous-stable and ready-to-use. With a colorant concentrate in the supplement, it is easy to differentiate between a Soleris vial with or without addition of supplement. As organisms grow in the broth medium, the carbon dioxide (CO_2_) produced diffuses through a membrane layer into a soft agar plug containing a dye indicator. CO_2_ released during organism growth changes the color of the agar plug from green or green-blue to yellow. The color change in the dye is read in real time by the Soleris instrument, and the system software denotes a positive detection time (Figure 1).

**Figure 1.**
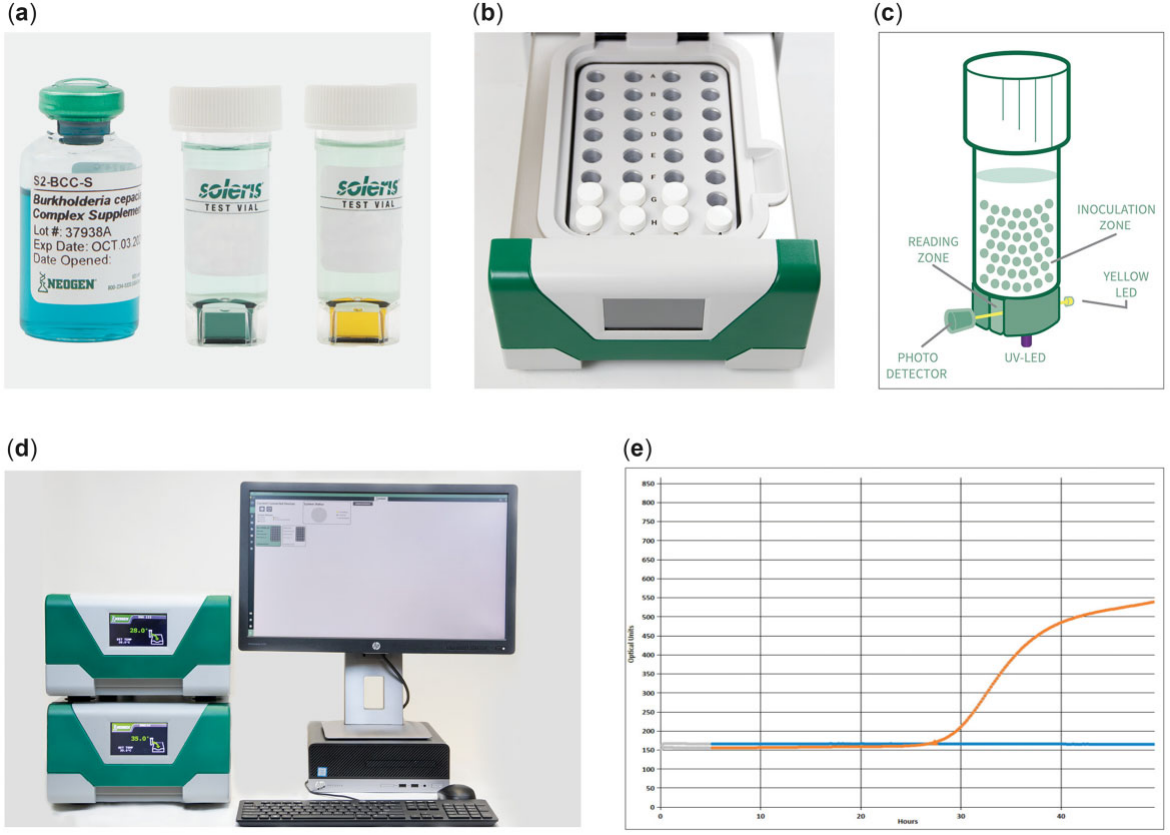
Soleris Bcc detection system. (a) Bcc supplement, S2-BCC-S (blue aqueous solution); vial media show negative (green-blue agar plug) and positive (yellow agar plug) detections. (b) The vial rack in the instrument draw. (c) Theory of detection. (d) Soleris Next Generation system—the instrument and the software. (e) Detection curves generated by the software; blue curve shows negative detection, orange curve shows positive detection.

The objective of this study was to evaluate the performance of the Soleris Bcc assay against the reference method USP <60> in 28 cosmetic products. Inclusivity, exclusivity, ruggedness, robustness, repeatability, and stability studies were also conducted with the Soleris Bcc assay.

## Experimental

### Apparatus and Reagents

- *Soleris Burkholderia cepacia complex test.*—Neogen Corp., Lansing, MI, Cat No. Soleris Direct *Pseudomonas* (9 mL), PD-109; *B. cepacia* complex supplement, S2-BCC-S.
- *Soleris Next Gen Complete System.*—Neogen Corp., Cat No. SNG-INS32CS.
- *Tryptic Soy Agar (TSA).*—Neogen Corp., Cat No. NCM0002.
- *Tryptic Soy Broth (TSB).—*Neogen Corp., Cat No. NCM0004.
- *Butterfield’s phosphate buffer*.—Neogen Corp., Cat No. BLX-BB9.
- *Tween-80.—*Neogen Corp., Cat No. NCM4081.
- *Modified Letheen Broth (MLB).—*Neogen Corp., Cat No. BLX-MLT.
- *Tryptone Azolectin Tween Broth (TAT).—*Neogen Corp., Cat No. NCM0091.
- *Burkholderia cepacia selective agar (BCSA).—*Neogen Corp., Cat No. NCM0209.

### Inoculum Preparation

One or two colonies of each culture from TSA stock plate were transferred to TSB and incubated at 35°C for 24–48 h. The culture suspension was decimally diluted in Butterfield’s phosphate buffer. The appropriate dilution/volume (50–100 µL) was used to achieve the target spiking levels for sample inoculation and plated on TSA to check the inoculum.

### Inclusivity and Exclusivity Study

Twenty-six strains of Bcc bacteria (Table 2) and 31 non-Bcc bacteria (Table 3) from the American Type Culture Collection (ATCC) and the Neogen Corp. Culture Collection were tested on Soleris Bcc method for specificity. The same inclusive and exclusive organisms were also tested with USP reference method.

**Table 1.** Soleris Bcc assay testing parameters

Test	Ignore	Resolution	Detection level	Duration	Temperature
Bcc	60	1	10	48 h	35°C

**Table 2.** Inclusivity study

Organism	Source^a^	Inoculum (CFU/90 mL)	MLB&T^b^		MLB^b^		TAT^b^	
			USP^c^	Soleris DT [h(SD)]^d^	USP^c^	Soleris DT [h(SD)]^d^	USP^c^	Soleris DT [h(SD)]^d^
*B. cenocepacia*	ATCC BAA-245	47	+	15.9 (0.1)	+	16.2 (0.6)	+	18.4 (0.4)
*B. cepacia*	ATCC 25416	34	+	5.8 (0.0)	+	5.8 (0.0)	+	5.8 (0.0)
*B. cenocepacia*	ATCC 25608	45	+	5.8 (0.0)	+	5.8 (0.0)	+	5.8 (0.0)
*B. multivorans*	ATCC BAA-247	51	+	5.8 (0.0)	+	5.8 (0.0)	+	5.8 (0.0)
*B. cepacia*	GT 853	70	+	7.8 (0.1)	+	6.9 (0.1)	+	5.8 (0.0)
*B. cepacia*	GT 1904	41	+	5.8 (0.0)	+	5.8 (0.0)	+	5.8 (0.0)
*B. cepacia*	GT 1905	30	+	5.8 (0.0)	+	5.8 (0.0)	+	5.8 (0.0)
*B. cepacia*	GT 1906	47	+	5.8 (0.0)	+	5.8 (0.0)	+	5.8 (0.0)
*B. cepacia*	GT 1907	30	+	5.8 (0.0)	+	5.8 (0.0)	+	5.8 (0.0)
*B. cepacia*	ATCC 13945	33	+	5.8 (0.0)	+	5.8 (0.0)	+	5.8 (0.0)
*B. cepacia*	GT 3120	42	+	5.8 (0.0)	+	5.9 (0.1)	+	5.8 (0.0)
*B. cepacia*	GT 3121	91	+	8.6 (0.6)	+	5.8 (0.0)	+	5.8 (0.0)
*B. cepacia*	GT 3122	13	+	5.8 (0.0)	+	5.8 (0.0)	+	5.8 (0.0)
*B. cepacia*	GT 3123	55	+	5.8 (0.0)	+	5.8 (0.0)	+	5.8 (0.0)
*B. cepacia*	GT 3124	48	+	6.0 (0.1)	+	5.8 (0.0)	+	5.8 (0.0)
*B. cepacia*	GT 3125	81	+	6.0 (0.0)	+	5.8 (0.0)	+	5.8 (0.0)
*B. cepacia*	GT 3126	105	+	5.8 (0.0)	+	5.8 (0.0)	+	5.8 (0.0)
*B. cepacia*	GT 3127	22	+	10.8 (0.0)	+	8.9 (0.1)	+	11.4 (0.1)
*B. cepacia*	ATCC 17774	29	+	6.4 (0.3)	+	5.9 (0.1)	+	5.8 (0.0)
*B. cepacia*	GT 3143	14	+	6.2 (0.1)	+	5.9 (0.0)	+	6.2 (0.3)
*B. cepacia*	GT 3144	37	+	7.1 (0.1)	+	6.8 (0.1)	+	5.8 (0.0)
*B. cepacia*	GT 3238	12	+	5.8 (0.0)	+	5.8 (0.0)	+	5.8 (0.0)
*B. cepacia*	GT 3239	46	+	5.8 (0.0)	+	5.8 (0.0)	+	5.8 (0.0)
*B. cepacia*	GT 3249	58	+	10.4 (0.1)	+	9.8 (0.2)	+	7.2 (0.1)
*B. cepacia*	GT 3520	13	+	30.6 (0.0)	+	35.8 (0.2)	+	43.7 (0.3)
*B. cepacia*	GT 3521	92	+	43.6 (0.7)	+	36.8 (1.1)	−	29.8 (0.3)

a ATCC = American Type Culture Collection; GT = Neogen in-house culture collection (Gene Track number).

b MLB&T = MLB with 10 g/L of Tween 80; MLB = Modified Letheen Broth; TAT = Tryptone Azolectin Tween Broth.

c Reference culture method following USP < 60>. +: positive result, –: negative result.

d Soleris Bcc mean detection time (h) with standard deviation of two replicates.

**Table 3.** Exclusivity study

Organism	Source^a^	Inoculum (CFU/90 mL)	MLB&T^b^		MLB^b^		TAT^b^	
			USP^c^	Soleris DT [h(SD)]^d^	USP^c^	Soleris DT [h(SD)]^d^	USP^c^	Soleris DT [h(SD)]^d^
*Acetobacter pasteurianus*	ATCC 12879	120	–	ND	–	ND	–	ND
*Bacillus subtilis*	ATCC 6633	320	–	ND	–	ND	–	ND
*Bacillus subtilis*	ATCC 9372	280	–	ND	–	ND	–	ND
*Burkholderia gladioli*	GT 3056	360	+	ND	+	ND	+	ND
*Burkholderia gladioli*	GT 3058	490	+	ND	+	ND	+	ND
*Burkholderia gladioli*	GT 3059	180	+	ND	+	ND	+	ND
*Burkholderia gladioli*	GT 3060	920	+	ND	+	ND	+	21.4 (0.1)
*Burkholderia gladioli*	GT 3061	1340	+	ND	+	ND	+	ND
*Burkholderia gladioli*	ATCC 51989	1220	+	ND	+	ND	+	ND
*Citrobacter freundii*	ATCC 8090	470	–	ND	–	ND	–	ND
*Enterobacter aerogenes*	ATCC 13048	840	–	ND	–	5.8 (0.1)	–	ND
*Enterobacter cloacae*	ATCC 23355	570	–	ND	–	ND	–	ND
*Enterobacter faecalis*	ATCC 19433	330	+	ND	+	5.8 (0.1)	–	ND
*Escherichia coli*	ATCC 25922	790	–	ND	–	ND	–	ND
*Escherichia coli*	ATCC 8739	1100	–	ND	–	ND	–	ND
*Klebsiella pneumoniae*	ATCC 13883	460	–	ND	–	ND	–	ND
*Proteus mirabilis*	ATCC 25933	940	+	ND	+	ND	+	ND
*Proteus vulgaris*	ATCC 8247	810	+	ND	+	ND	+	ND
*Pseudomonas aeruginosa*	ATCC 9027	650	–	ND	–	ND	–	ND
*Pseudomonas aeruginosa*	ATCC 27853	550	–	ND	–	ND	+	ND
*Pseudomonas aeruginosa*	ATCC 10145	400	–	ND	–	ND	–	ND
*Salmonella enteritidis*	ATCC 13076	540	–	ND	–	ND	–	ND
*Serratia marcescens*	ATCC 13880	380	+	ND	+	ND	+	ND
*Serratia marcescens*	ATCC 8100	410	+	ND	+	ND	+	ND
*Staphylococcus aureus*	ATCC 6538	340	–	ND	–	ND	–	ND
*Staphylococcus aureus*	ATCC 25923	510	–	ND	–	ND	–	ND
*Staphylococcus epidermidis*	ATCC 12228	420	–	ND	–	ND	–	ND
*Stenotrophomonas maltophilia*	GT 3252	450	+	ND	+	ND	+	ND
*Stenotrophomonas maltophilia*	GT 3253	570	+	ND	–	ND	+	ND
*Stenotrophomonas maltophilia*	ATCC 51331	770	+	ND	+	ND	+	15.9 (0.3)
*Stenotrophomonas maltophilia*	ATCC 13637	790	–	ND	–	ND	+	ND

a ATCC = American Type Culture Collection; GT = Neogen in-house culture collection (Gene Track number).

b MLB&T: MLB with 10 g/L of Tween 80; MLB = Modified Letheen Broth; TAT = Tryptone Azolectin Tween Broth.

c Reference culture method following USP < 60>. +: positive result, –: negative result.

d Solieris Bcc method mean detection time (h) with standard deviation of two replicates. ND: no detection.

For the inclusivity study, 50 µL of the diluted culture suspension was inoculated into 90 mL of pre-enrichment broth to achieve a target spiking concentration of 10–100 CFU. Three pre-enrichment (neutralizing) broths were inoculated in parallel: *(1)* MLB, *(2)* TAT, and *(3)* Modified Letheen Broth with 10 g/L of Tween 80 (MLB&T).

**Figure 2.**
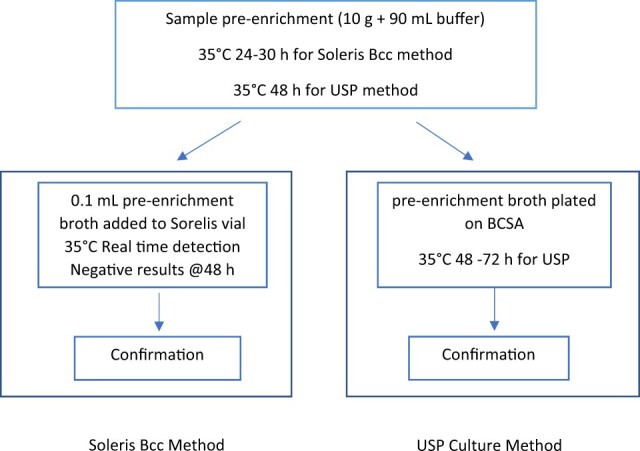
Flowchart of detection of Bcc with Soleris and USP method.

For the exclusivity study, similar procedures were followed, except the target inoculum was 100–1000 CFU in 90 mL of each of the neutralizing broth. Inclusivity and exclusivity organisms were tested with both the Soleris Bcc method and the USP reference method (Figure 2).

For the Soleris Bcc method, the inclusivity and exclusivity samples were incubated at 35°C for 24–30 h. After pre-enrichment, 0.1 mL aliquots were transferred to Soleris PD-109 vials supplemented with 0.2 mL of Bcc supplement (S2-Bcc-S), mixed well, placed into the Soleris instrument, and tested with the parameters listed in Table 1. Duplicate vials were tested for each sample.

For the USP reference method, the inclusivity and exclusivity samples were incubated at 35°C for 48 h, and then subcultured onto BCSA and incubated the plates at 35°C for 48–72 h (24).

### Matrix Study

Cosmetic products were purchased from local stores. Twenty-eight cosmetic matrixes included petroleum jelly, hydrocortisone cream, toothpaste, shave gel, makeup remover, concealer, hair mousse, 2-in-1 shampoo and conditioner, baby shampoo, baby lotion, ointment, body and face lotion for men, hand soap, hand cream, sunscreen lotion SPF 15, finishing powder, face/neck cream, lip balm brand A, hair putty, mousse foundation, lipstick brand A, eye pencil, lip balm brand B, body lotion, lipstick brand B, pressed powder, face mask, and after-sun aloe vera lotion.

Matrix testing samples were prepared by aseptically weighing 10 g of well-mixed cosmetic product into a sterile container, combined with 90 mL of one of the three pre-enrichment broths, *(1)* MLB; *(2)* TAT, *(3)* MLB&T, a 1:1 ratio of a product matrix was prepared by adding 10 g of product to 10 g of Tween 80, mixing well, and allowing it to neutralize for 30 min before adding to 80 mL of MLB (MLB&T).

Six different Bcc organisms were tested in the matrix trials including a total of 28 cosmetic products. As per USP testing guidelines for challenge studies, the diluted culture suspension (0.1 mL) targeting at 1–5 CFU/g was inoculated to the Product/Broth mixture, which was neutralized for 30 min.

For the Soleris Bcc method, both unspiked and spiked samples were incubated at 35°C for 24–30 h, 0.1 mL of pre-enrichment aliquots were transferred to Soleris PD-109 vials supplemented with 0.2 mL of Bcc supplement (S2-Bcc-S), and the same procedures were followed as described in the inclusivity and exclusivity study. Duplicates were tested for each sample. Then the Soleris vial samples were streaked onto BCSA for confirmation.

For the USP reference method, both unspiked and spiked samples were incubated at 30 to 35°C for 48–72 h, and then subcultured onto BCSA. The plates were incubated at 30 to 35°C for 48–72 h and with presumptive positive results were further confirmed by identification tests.

### Repeatability

Four cosmetic products, namely makeup remover, petroleum jelly, hand cream, and ointment, were tested to evaluate the degree of agreement among individual test results. *B. cepacia* ATCC 25416, *B. cenocepacia* ATCC BAA-245, *B. cenocepacia* ATCC 25608, and *B. multivorans* ATCC BA-247 were used to inoculate the products.

The product matrix was prepared by adding 90 mL of MLB to 10 g of product. The product/MLB mixture was inoculated with 0.1 mL of diluted Bcc culture to achieve a target spike concentration of 5–10 CFU/g. The samples were incubated at 35°C for 24–30 h. Twenty replicates were tested for each matrix with the Soleris Bcc assay.

### Product Robustness, Lot-to-Lot Consistency Study

The effect of modest perturbations to Soleris operating parameters were tested, including incubation temperatures, sample sizes, and media volumes. The degree of precision of the test results was also evaluated with different analysts and instruments. Additionally, lot-to-lot reagent consistency was assessed. Each parameter was tested using four different strains of *Burkholderia* with six replicates (Table 4). The TSB (90 mL) was inoculated with 0.1 mL of diluted Bcc culture to achieve a target spike concentration of 10–100 CFU per sample. The procedures for the Soleris Bcc assay described previously were followed.

**Table 4.** Parameters tested in the ruggedness and robustness study

Parameters	Variables
Instrument temperature	34.5°C, 35°C, 35.5°C
Sample size	90 µL, 100 µL, 110 µL
Media volume	8.5 mL, 9.0 mL, 9.5 mL
Analyst	Analyst A, B, C
Instrument	Instrument A, B, C
Lot-to-lot	Same base vial lot, different supplement lots (A, B) Different base vial lots, same supplement lot (C, D)
Organisms	*B. cepacia* ATCC 25416, *B. cenocepacia* ATCC BAA-245
	*B. cenocepacia* ATCC 25608, *B. multivorans* ATCC BAA-247

### Stability and Simulated Shipping Study

For the stability study, a single lot of Bcc supplement was stored at 2–8°C and 35°C for up to 9 weeks. Inclusivity and exclusivity of Bcc organisms were tested with the Bcc supplement stored at different conditions.

To simulate the temperature abuse during shipping, three lots of Bcc supplement were placed at 35°C for 7 days, transferred to 2–8°C for 7 days, and then transferred to 35°C for 7 days again before testing. Three Bcc bacteria (*B. cepacia* ATCC 25416, *B. cenocepacia* ATCC BAA-245, and *B. multivorans* ATCC BAA-247) and one non-Bcc organism (*Stenotrophomonas maltophilia* ATCC 13637) were tested using simulated shipped and control media with 10 replicates. The TSB (90 mL) was inoculated with 10–100 CFU of Bcc culture and 100–1000 CFU of non-Bcc organism. To verify testing conditions, specifically that there was no contamination during the testing workflow, a negative control was included using the chosen diluent (TSB) in place of the test sample. The procedures for the Soleris Bcc assay were followed as described previously.

### Data Analysis

To compare the method equivalency in the matrix study, a two-row by two-column contingency table with respect to the reference culture method and Soleris Bcc vial method was constructed. Inclusivity, exclusivity, positive predictivity, negative predictivity, analytical accuracy, and Kappa index were calculated (25). For the repeatability study, the coefficient of variation was calculated among 20 replicates for each of the product and challenge organism combinations (26, 27). For the robustness and ruggedness study, a one-way analysis of variance (ANOVA) was performed to determine if there were significant differences between the data sets for each parameter. A *t*-test was performed to determine if there were significant differences between the non-shipped and shipped vials. The statistical analysis was performed on the combined data from all three lots of materials for each organism. A *P*-value <0.05 indicates statistically different results as the 5% level of significance (α = 0.05). All statistical analyses were performed using Excel and Mini-Tab.

## Results and Discussion

### Inclusivity and Exclusivity Study

Inclusivity and exclusivity studies were performed to evaluate the ability of the Soleris Bcc assay to support the growth of Bcc bacteria and inhibit the growth of non-Bcc organisms. A total of 26 Bcc strains were tested in the inclusivity study using three different pre-enrichment broths. Both Soleris Bcc test and USP reference culture methods were able to detect all Bcc strains through all three pre-enrichment broths, except the USP method showed negative results with *B. cepacia* GT 3120 in TAT (Table 2).

Cosmetics contain preservatives, so it is critical to neutralize the preservatives to enable any viable cells to resuscitate and proliferate in the culture media. However, cosmetics are made with different formulas and a diverse mix of preservatives. A universal broth capable of neutralizing the antibacterial activity from a wide variety of products has been unsuccessful (28, 29). TAT broth and MLB are two commonly used neutralizing broths for microbiological testing of cosmetic products. TAT is included in ISO 21149 for enumeration and detection of aerobic mesophilic bacteria in cosmetics (30) and is recommended by the USP for microbial examinations of nonsterile products (29). MLB is used as a pre-enrichment medium, neutralizer, and diluent for the isolation of most microorganisms from cosmetic samples by U.S. Food and Drug Administration *Bacteriological Analytical Manual* (30). In addition to neutralizing agents, TAT and MLB contain ingredients that provide nutrients required for the growth of a wide variety of microorganisms. Extra Tween 80 to MLB was also included as the pre-enrichment broth (MLB&T). This has been shown to be helpful with solids/powders and cream/oil-based cosmetic products (29, 31).

The exclusivity panel consisted of a total of 31 bacteria, including six strains belonging to *Burkholderia* species that do not belong to Bcc. No false positives were observed in MLB&T enrichment with the Soleris assay, while the USP reference method detected 14 out of 31 (45%). Only two non-Bcc strains tested as positive from the MLB and TAT Broth with Soleris assay, while the USP reference method resulted in 13 and 15 positives (Table 3). The Soleris Bcc test did not detect most of the exclusive organisms, as expected, achieving better specificity performance compared to the USP reference *Burkholderia cepacia* Selective Agar method.

### Matrix Study to Compare Method Equivalency

To validate the Soleris Bcc test, a two-fold approach was considered: (1) paired sample analysis of unspiked product matrixes, and *(2)* paired sample analysis of product matrixes spiked with low levels of challenge organisms by both the Soleris Bcc test and compendial plating method.

A total of 56 product matrixes (28 unspiked and 28 spiked) were assessed by both the Soleris Bcc and the USP plating method. For the unspiked matrixes, both the Soleris Bcc and reference method showed negative results (Supplemental Table S1). For the spiked products, all the 28 spiked samples analyzed via the Soleris Bcc test displayed growth as expected; however, the USP reference method missed one sample—baby shampoo, which was inoculated with a low number of *Burkholderia cenocepacia* ATCC BAA-245 (Supplemental Table S2). A two-by-two contingency table with respect to the USP reference method and Soleris Bcc is shown in Supplemental Table S3.

Compared to the USP culture method, the Soleris Bcc assay shows 100% diagnostic sensitivity and 96.6% specificity. The positive predictive value was 96.4%, the negative predictive value was 100%, the analytical accuracy value was 0.98, and the Kappa index was 0.96 (Table 5), indicating the Soleris Bcc test shows equivalent results as the reference USP plating method.

**Table 5.** Matrix study: Statistics for Soleris Bcc method

Sensitivity	100%
Specificity	96.6%
Positive predictivity, %	96.4%
Negative predictivity, %	100%
Analytical accuracy	0.98
Kappa index	0.96

### Repeatability

The repeatability study consisted of four unique product matrixes being analyzed with the Soleris Bcc test. The matrixes were spiked with a low number of challenge organisms, and 20 replicates were assessed. The coefficient of variation was 0.0, 1.9, 0.0, and 2.8% for makeup remover spiked with *Burkholderia cepacia* ATCC 25416, petroleum jelly spiked with *Burkholderia cenocepacia* ATCC BAA-245*,* hand cream spiked with *Burkholderia cenocepacia* ATCC 25608, and healing ointment spiked with *Burkholderia multivorans* ATCC BAA-247, respectively (Table 6). The results demonstrate that the Soleris Bcc test shows precise results.

**Table 6.** Soleris Bcc repeatability test result

Matrix	Organism	Detection time, h^a^	Standard deviation	Coefficient of variation, %
Makeup remover	*B*. *cepacia*	5.8	0.0	0.0
	ATCC 25416			
Petroleum jelly	*B*. *cenocepacia*	23.1	0.5	1.9
	ATCC BAA-245			
Hand cream	*B*. *cenocepacia*	5.8	0.0	0.0
	ATCC 25608			
Ointment	*B. multivorans*	7.4	0.2	2.8
	ATCC BAA-247			

a Values are mean detection time from 20 replicates.

### Product Robustness, Consistency (Lot-to-Lot), and Stability Studies

Bcc comprises more than 20 closely related species (5, 32). The USP < 60> specifies three strains for growth promotion test, namely *Burkholderia cepacia* ATCC 25416*, Burkholderia cenocepacia* BAA-245, and *Burkholderia multivorans* ATCC BAA-247, because these three members from Bcc are the most clinically significant in cystitis fibrosis infections.

The same detection times were observed with moderate variations of incubation temperature, sample size, media volume, analyst, instrument, and lot-to-lot of reagents for detection of *B. cepacia* ATCC 25416, *B. multivorans* ATCC BAA-247, and *B. cenocepacia* ATCC 25608, compared to the standard test conditions (Table 7). No significant difference (*P *>* *0.05) in detection times was observed for detection of *B. cenocepacia* ATCC BAA-245 by different analysts or using four different lots of Bcc reagents. Slight differences in detection time were found with the variation of temperature and media volume and instrument units for detection of *B. cenocepacia* ATCC BAA-245; however, the difference was minimal, all within 1 h. The growth rate of *B. cenocepacia* ATCC BAA-245 was not as rapid when compared to other Bcc organisms. The Soleris Bcc method detected it within 21 h, and the other three strains of Bcc were detected within 6 h, all with the inoculum in the range of 10–100 CFU.

**Table 7.** Summary of robustness study for Soleris Bcc assay

Parameters	Variables	Soleris Bcc detection time [h (standard deviation)]^a^			
		*B. cepacia*	*B. cenocepacia*	*B. multivorans*	*B. cenocepacia*
		ATCC 25416	ATCC BAA-245	ATCC BAA-247	ATCC 25608
Temperature, °C	34.5	5.8 (0.0)^x^	20.5 (0.3)^x^	5.8 (0.0)^x^	5.8 (0.0)^x^
	35.0	5.8 (0.0)^x^	20.5 (0.4)^x^	5.8 (0.0)^x^	5.8 (0.0)^x^
	35.5	5.8 (0.0)^x^	19.9 (0.5)^y^	5.8 (0.0)^x^	5.8 (0.0)^x^
Sample size, µL	90	5.8 (0.0)^x^	20.5 (0.4)^x^	5.8 (0.0)^x^	5.8 (0.0)^x^
	100	5.8 (0.0)^x^	20.7 (1.0)^x^	5.8 (0.0)^x^	5.8 (0.0)^x^
	110	5.8 (0.0)^x^	20.3 (0.7)^x^	5.8 (0.0)^x^	5.8 (0.0)^x^
Media volume, mL	8.5	5.8 (0.0)^x^	21.2 (0.2)^x^	5.8 (0.0)^x^	5.8 (0.0)^x^
	9.0	5.8 (0.0)^x^	20.5 (0.4)^y^	5.8 (0.0)^x^	5.8 (0.0)^x^
	9.5	5.8 (0.0)^x^	20.3 (0.3)^y^	5.8 (0.0)^x^	5.8 (0.0)^x^
Analyst	A	5.8 (0.0)^x^	11.9 (0.4)^x^	5.8 (0.0)^x^	5.8 (0.0)^x^
	B	5.8 (0.0)^x^	11.8 (0.2)^x^	5.8 (0.0)^x^	5.8 (0.0)^x^
	C	5.8 (0.0)^x^	12.1 (0.3)^x^	5.8 (0.0)^x^	5.8 (0.0)^x^
Instrument	A	5.8 (0.0)^x^	11.5 (0.2)^x^	5.8 (0.0)^x^	5.8 (0.0)^x^
	B	5.8 (0.0)^x^	11.5 (0.2)^x^	5.8 (0.0)^x^	5.8 (0.0)^x^
	C	5.8 (0.0)^x^	12.1 (0.3)^y^	5.8 (0.0)^x^	5.8 (0.0)^x^
Lot-to-lot	A	5.8 (0.0)^x^	20.6 (0.4)^x^	5.8 (0.0)^x^	5.8 (0.0)^x^
	B	5.8 (0.0)^x^	20.5 (0.4)^x^	5.8 (0.0)^x^	5.8 (0.0)^x^
	C	5.8 (0.0)^x^	20.3 (0.5)^x^	5.8 (0.0)^x^	5.8 (0.0)^x^
	D	5.8 (0.0)^x^	20.3 (0.3)^x^	5.8 (0.0)^x^	5.8 (0.0)^x^

a Values are mean detection time from six replicates and standard deviation. Means followed by a common letter within the same parameter are not significantly different by the ANOVA at the 5% level of significance.

The test assay reagent stability study showed that after 9 weeks of storage, Bcc detection times were similar among the vials stored at refrigeration temperature (2–8°C), accelerated stability simulated with higher storage temperature (35°C), and freshly made reagents (data not shown).

A simulated shipping study was performed to determine if shipping conditions impacted the recovery of Bcc and the detection time, and the selectivity of the medium was maintained to inhibit the exclusive organisms. The Bcc vials were placed at 35°C for 7 days to represent the high temperatures that may occur during transportation in a warm climate without refrigeration. Then the Bcc vials were transferred from 35°C storage and placed at 2–8°C for 7 days to represent storage at a distributor prior to shipping to one of their local customers. Lastly, the Bcc vials were transferred from 2–8°C storage to 35°C storage for 7 days to represent the high temperatures that may occur during transportation from the distributor to the customer without refrigeration. Results from the simulated shipping study show that Bcc detection times were not significantly different (*P *>* *0.05) between the simulated shipping vials and control vials (Table 8).

**Table 8.** Simulated shipping study: Soleris Bcc detection time of control and simulated shipping vials

	Control vials [h (standard deviation]^a^	Simulated shipped vials [h (standard deviation)]^a^
*B. cepacia* ATCC 25416	5.8 (0.0)	5.8 (0.0)
*B. cenocepacia* ATCC BAA-245 ^b^	12.8 (0.2)	12.8 (0.3)
*B. multivorans* ATCC BAA-247	5.8 (0.0)	5.8 (0.0)
*S. maltophilia* ATCC 13637	ND ^c^	ND
Negative Control (TSB)	ND	ND

a Values are mean detection time from 10 replicates and standard deviation.

b *T*-test showed no significant difference (*P *=* *0.456) between the control and simulated shipped vials.

c ND = No detection.

## Conclusions

The Soleris Bcc assay was developed and validated for detecting *B. cepacia* complex bacteria in cosmetic products. The results show equivalent or better performance of the Soleris Bcc method compared to the USP reference method. The ready-to-use Soleris Bcc method provides a rapid detection solution for Bcc in cosmetic products. Furthermore, this method minimizes sample handling steps and provides automated real-time results within 48 h after pre-enrichment. The newly developed Soleris Bcc method allows customers to rapidly detect *Burkholderia cepacia* complex organisms in personal care products.

## Conflict of Interest

The authors declare that they have no conflict of interest.

## Supplemental Information

Supplemental information is available on the *J. AOAC Int*. website.

## Supplementary Material

qsac109_Supplementary_Data
