# Supplementary material for: Soleris® Automated System for the Rapid Detection of Burkholderia cepacia Complex in Cosmetic Products
Source: J AOAC Int. 2022 Sep 21;106(1):171–8. doi: 10.1093/jaoacint/qsac109 (PMC9779911; doi:10.1093/jaoacint/qsac109)
Supplement: qsac109_Supplementary_Data [file qsac109_supplementary_data.zip › suppl_data/aoac-22-0130-File014.docx]

Supplemental Table 3. Two-by-two contingency table to compare USP reference method and Soleris Bcc method

| Soleris Bcc method | USP method | | Row Totals |
| --- | --- | --- | --- |
|  | Positive | Negative |  |
| Positive | 27 | 1 | 28 |
| Negative | 0 | 28 | 28 |
| Column Totals | 27 | 29 | 56 |
