# Supplementary material for: Soleris® Automated System for the Rapid Detection of Burkholderia cepacia Complex in Cosmetic Products
Source: J AOAC Int. 2022 Sep 21;106(1):171–8. doi: 10.1093/jaoacint/qsac109 (PMC9779911; doi:10.1093/jaoacint/qsac109)
Supplement: qsac109_Supplementary_Data [file qsac109_supplementary_data.zip › suppl_data/aoac-22-0130-File013.docx]

Supplemental Table 2. Matrix study – products spiked with low level (1-5 CFU/g) of challenge organisms

| Matrix | Organism | Source | Pre- enrich-ment broth^a^ | Soleris Bcc Method | | USP Ref. Method (+/-) | Agreement Between Methods |
| --- | --- | --- | --- | --- | --- | --- | --- |
|  |  |  |  | Detection Time [h(SD)] ^b^ | Confirmation on BCSA (+/-)^c^ |  |  |
| Petroleum Jelly | *Burkholderia cepacia* | ATCC 25416 | TAT | 16.4 (0.0) | + | + | Yes |
| Hydrocortisone Cream 1% | *Burkholderia cepacia* | ATCC 25416 | MLB | 16.4 (0.0) | + | + | Yes |
| Fresh Mint Sensitive Toothpaste | *Burkholderia cepacia* | ATCC 25416 | TAT | 16.4 (0.0) | + | + | Yes |
| Sensitive Skin Shave Gel | *Burkholderia cepacia* | ATCC 25416 | MLB | 25.9 (3.3) | + | + | Yes |
| Makeup Remover | *Burkholderia cepacia* | ATCC 25416 | MLB | 5.8 (0.0) | + | + | Yes |
| Concealer | *Burkholderia cepacia* | ATCC 25416 | MLB&T | 5.8 (0.0) | + | + | Yes |
| Mousse | *Burkholderia cenocepacia* | ATCC BAA-245 | TAT | 30.9 (0.4) | + | + | Yes |
| 2 in 1 Shampoo & Conditioner | *Burkholderia cenocepacia* | ATCC BAA-245 | MLB | 24.8 (0.0) | + | + | Yes |
| Baby Shampoo | *Burkholderia cenocepacia* | ATCC BAA-245 | MLB&T | 42.1 (0.8) | + | - | **No** |
| Baby Lotion | *Burkholderia cenocepacia* | ATCC BAA-245 | MLB | 25.4 (0.4) | + | + | Yes |
| Ointment | *Burkholderia multivorans* | ATCC BAA-247 | MLB | 5.8 (0.0) | + | + | Yes |
| Body and Face Lotion for Men | *Burkholderia multivorans* | ATCC BAA-247 | MLB | 10.0 (0.2) | + | + | Yes |
| Hand Soap | *Burkholderia multivorans* | ATCC BAA-247 | MLB&T | 6.1 (0.4) | + | + | Yes |
| Hand Cream | *Burkholderia multivorans* | ATCC BAA-247 | TAT | 5.8 (0.0) | + | + | Yes |
| Sunscreen Lotion SPF 15 | *Burkholderia multivorans* | ATCC BAA-247 | TAT | 9.6 (0.1) | + | + | Yes |
| Finishing Powder | *Burkholderia cenocepacia* | ATCC 25608 | TAT | 5.8 (0.0) | + | + | Yes |
| Face/Neck Cream | *Burkholderia cenocepacia* | ATCC 25608 | TAT | 5.8 (0.0) | + | + | Yes |
| Raspberry Lip Balm | *Burkholderia cenocepacia* | ATCC 25608 | TAT | 11.5 (0.6) | + | + | Yes |
| Surfer Hair Power Putty | *Burkholderia cenocepacia* | ATCC 25608 | MLB&T | 11.9 (0.5) | + | + | Yes |
| Mousse Foundation | *Burkholderia cepacia* | ATCC 17774 | TAT | 10.0 (0.4) | + | + | Yes |
| Lipstick A | *Burkholderia cepacia* | ATCC 17774 | MLB&T | 9.4 (0.1) | + | + | Yes |
| Eye Pencil | *Burkholderia cepacia* | ATCC 17774 | TAT | 13.5 (0.0) | + | + | Yes |
| Orange Mango Lip Balm | *Burkholderia cepacia* | ATCC 17774 | TAT | 14.7 (0.8) | + | + | Yes |
| Body Lotion | *Burkholderia cepacia* | GT 853 | MLB | 33.8 (10.5) | + | + | Yes |
| Lip Stick B | *Burkholderia cepacia* | GT 853 | MLB | 23.4 (1.7) | + | + | Yes |
| Pressed Powder | *Burkholderia cepacia* | GT 853 | MLB | 7.6 (0.1) | + | + | Yes |
| Brightening Face Mask | *Burkholderia cepacia* | GT 853 | TAT | 16.4 (0.0) | + | + | Yes |
| Aloe Vera After Sun Lotion | *Burkholderia cepacia* | GT 853 | MLB&T | 16.4 (0.0) | + | + | Yes |

^a^ Three enrichment broths were used. TAT: Tryptone Azolectin Tween Broth; MLB: Modified Letheen Broth; MLB&T:

10 g Tween 80 added to 10 g product neutralized for 30 minutes and then adding 90 mL of MLB.

^b^ Soleris Bcc mean detection time (h) with standard deviation of two replicates

^c^ Both vials were streaked on BCSA for confirmation and showed the same results.
