# Supplementary material for: Soleris® Automated System for the Rapid Detection of Burkholderia cepacia Complex in Cosmetic Products
Source: J AOAC Int. 2022 Sep 21;106(1):171–8. doi: 10.1093/jaoacint/qsac109 (PMC9779911; doi:10.1093/jaoacint/qsac109)
Supplement: qsac109_Supplementary_Data [file qsac109_supplementary_data.zip › suppl_data/aoac-22-0130-File012.docx]

Supplemental Table 1. Matrix study – un-spiked products

| Matrix | Pre-Enrichment Broth^a^ | Soleris Bcc method | | USP Ref. Method  (+/-) | Agreement Between Methods |
| --- | --- | --- | --- | --- | --- |
|  |  | Detection Time (h)^b^ | Confirmation on BCSA (+/-)^c^ |  |  |
| Petroleum Jelly | TAT | ND | - | - | Yes |
| Hydrocortisone Cream 1% | MLB | ND | - | - | Yes |
| Fresh Mint Sensitive Toothpaste | TAT | ND | - | - | Yes |
| Sensitive Skin Shave Gel | MLB | ND | - | - | Yes |
| Makeup Remover | MLB | ND | - | - | Yes |
| Concealer | MLB&T | ND | - | - | Yes |
| Mousse | TAT | ND | - | - | Yes |
| 2 in 1 Shampoo & Conditioner | MLB | ND | - | - | Yes |
| Baby Shampoo | MLB&T | ND | - | - | Yes |
| Baby Lotion | MLB | ND | - | - | Yes |
| Ointment | MLB | ND | - | - | Yes |
| Body and Face Lotion for Men | MLB | ND | - | - | Yes |
| Hand Soap | MLB&T | ND | - | - | Yes |
| Hand Cream | TAT | ND | - | - | Yes |
| Sunscreen Lotion SPF 15 | TAT | ND | - | - | Yes |
| Finishing Powder | TAT | ND | - | - | Yes |
| Face/Neck Cream | TAT | ND | - | - | Yes |
| Raspberry - Lip Balm | TAT | ND | - | - | Yes |
| Surfer Hair Power Putty | MLB&T | ND | - | - | Yes |
| Mousse Foundation | TAT | ND | - | - | Yes |
| Lipstick A | MLB&T | ND | - | - | Yes |
| Eye Pencil | TAT | ND | - | - | Yes |
| Orange Mango Lip Balm | TAT | ND | - | - | Yes |
| Body Lotion | MLB | ND | - | - | Yes |
| Lip Stick B | MLB | ND | - | - | Yes |
| Pressed Powder | MLB | ND | - | - | Yes |
| Brightening Face Mask | TAT | ND | - | - | Yes |
| Aloe Vera After Sun Lotion | MLB&T | ND | - | - | Yes |

^a^ TAT: Tryptone Azolectin Tween Broth; MLB: Modified Letheen Broth; MLB&T: 10 g Tween 80 added to 10 g product

neutralized for 30 minutes and then adding 90 mL of MLB.

^b^ Soleris Bcc method: Duplicates samples were tested and showed the same results. ND: no detection.

^c^.Both vials were streaked on BCSA for confirmation and showed the same results.
